# Stoichiometry of natural bacterial assemblages from lakes located across an elevational gradient

**DOI:** 10.1038/s41598-017-06282-0

**Published:** 2017-07-19

**Authors:** Birgit Stenzel, Carina Rofner, Maria Teresa Pérez, Ruben Sommaruga

**Affiliations:** University of Innsbruck, Institute of Ecology, Technikerstraße 25, 6020 Innsbruck, Austria

## Abstract

Heterotrophic bacteria are thought to be phosphorus-rich organisms with relatively homeostatic stoichiometry, but the elemental composition of natural bacterial communities has rarely been assessed. Here we tested whether bacterial stoichiometry changes with the trophic status of lakes by assessing the elemental composition of the bacterial-dominated (hereafter microbial) fraction together with that of the dissolved and seston fractions in 11 lakes situated along an elevational gradient. The stoichiometry of these three size-fractions was analyzed during the thermal stratification and mixing periods in composite water samples and in the water layer of the deep chlorophyll-a maximum. In addition, we analyzed the relative abundance of the most common bacterial groups in the lakes. Our results show that the microbial fraction was always enriched in phosphorus compared to the dissolved fraction, irrespectively of the lake trophic status. Further, they indicate that the elemental composition of bacteria in mountain lakes is at least seasonally very dynamic, resulting not only from changes in the nutrient ratios of the resource itself, but probably from changes in the composition of the dominant bacterial taxa too, though at the taxonomic level analyzed, we did not find evidence for this.

## Introduction

Bacteria in aquatic ecosystems fulfill important ecosystem functions such as processing allochthonous and autochthonous organic matter^1, 2^ and therefore are key in the cycling of carbon, nitrogen and phosphorus^1^. Besides transforming dissolved organic matter (DOM) into particulate biomass, bacteria can mineralize DOM into nutrients^3–5^. However, the classical view of heterotrophic bacteria as mineralizers has long been challenged by the fact that they can take up inorganic nutrients at high rates^4, 6^ and compete for limiting nutrients such as phosphorus with phytoplankton^4, 7, 8^. Ecological stoichiometry theory offers an alternative way to address the “sink or link” controversy by considering that bacteria will act as sink or link of nutrients depending on their own stoichiometry and on that of the available resources^9, 10^. Thus, if the nutrient content in bacteria is higher than that of the dissolved fraction, they will immobilize nutrients, whereas if it is lower, they will release or regenerate them^10^.

In aquatic environments, heterotrophic bacteria are generally considered to be exceptionally nutrient-rich, particularly in phosphorus, as compared to other compartments of the food web^11^. This assumption is based either on tracer uptake experiments that indicated bacteria dominate nutrient uptake at low nutrient concentrations^12–14^ or on direct measurements of the elemental composition of bacterial biomass^15^. Most of the measurements, however, have been done on cultures, due to the difficulty of separating heterotrophic bacteria from small autotrophs, and have mainly been focused on carbon and nitrogen. Whereas the C:N ratio is considered to be relatively constant among different bacteria and growth conditions^16^, the C:P and N:P ratios exhibit high variability^10^. Despite the importance that bacterial stoichiometry might have for the cycling of organic matter, the elemental composition of natural bacterial communities has rarely been assessed. Cotner *et al*.^10^ found that the stoichiometry of aquatic bacteria is flexible and that their elemental composition is very similar to that of the seston in samples collected from over 120 lakes in the USA. They also found that some bacteria are extremely nutrient poor (C:N:P = 875:179:1).

The use of cultivation-free methods to assess the diversity of microbial cells has become an important tool in microbial ecology^17^. In parallel, the combination of single-cell methods allowing taxonomical identification and the assessment of individual bacterial cell properties has also been largely used^18–20^. However, a method that allows for assessing simultaneously the taxonomic affiliation and the stoichiometry of bacterial cells is lacking. A central idea in biological stoichiometry is the growth rate hypothesis (GRH). The GRH postulates that faster growing organisms have an increasing demand on P to be allocated to the P-rich ribosomal RNA^21, 22^. Thus, fast growing organisms are more P-rich than slow growing ones. Indeed, different freshwater bacterial groups grow at very different rates^23^ and it is plausible that that this theory also applies to bacteria. For example, *Betaproteobacteria* and particularly the R-BT subgroup (see also *Limnohabitants*) grow faster than other bacterial groups^23^.

Here we assessed the stoichiometry of the microbial (0.7–1 µm) fraction in lakes located along an elevational gradient (567–2799 m above sea level) during the summer stratification period, when all lakes exhibited a well-developed deep chlorophyll-a maximum, and in autumn, when most lakes have already undergone mixis. For comparison, we also analyzed the stoichiometry of the dissolved (<0.7 µm) and seston (0.7–50 µm) fractions. We collected composite samples from the whole water column (CWS) and additionally, from the deep chlorophyll-a maximum (DCM). Though the presence of phototrophic bacteria in the microbial fraction cannot be completely excluded, autotrophic picoplankton in mountain lakes are either present in very low abundances or are absent^24^. Thus, we hypothesized that the elemental composition of the microbial fraction dominated by heterotrophic bacteria will differ among lakes of different trophic status (e.g., based on DOM and total phosphorus concentrations) as found along an elevational gradient^25, 26^. In other words, we expected that the microbial fraction will be more depleted in nutrients (N and P) relative to carbon in the more oligotrophic lakes situated higher in the elevational gradient. Additionally, we assessed the bacterial community composition to test whether in lakes dominated by the fast growing R-BT subgroup of *Betaproteobacteria*, the stoichiometry of the microbial fraction is more P-rich than in lakes where other taxonomic groups dominate the community.

## Results

### Stoichiometry of the dissolved fraction

Nutrient concentrations were always lower in water samples collected in lakes above the treeline than in those from lakes below it (Supplementary Table S1 and Table S2). TDP concentrations in July ranged from 0.30 to 5.30 µg L^−1^ and from 0.80 to 4.40 µg L^−1^ in October. The highest DOC concentration (5157 µg L^−1^) was measured in the CWS from Lansersee (LAS) and the lowest one (237 µg L^−1^) in Oberer Plenderlesee (OPL). TDN concentrations showed a large variability ranging from 50.02 µg L^−1^ in Schwarzsee ob Sölden (SOS) to 4329.07 µg L^−1^ in LAS (Supplementary Table S2 **)**.

In the dissolved fraction, the C:P ratio was always higher than the Redfield ratio (Table 1) and this pattern was more pronounced in lakes situated at low elevation. The C:N ratios were generally lower than the Redfield ratio (6.6). Three of the subalpine lakes had higher C:N ratios than the average (1.5) and two of them had a C:N higher than the Redfield one (Table 1).

**Table 1:** Summary of the stoichiometry of the dissolved fraction in composite water samples (CWS) and in samples from the deep chlorophyll-a maximum (DCM).

Lake	Elevation (m a.s.l)	Date	Sample	C:P	C:N	N:P	Date	Sample	C:P	C:N	N:P
SOS	2799	na	na	na	na	na	14.10.2013	DCM	962	7.66	125
GKS	2413	25.07.2013	DCM	1175	1.68	701	01.10.2013	DCM	756	2.29	330
OPL	2344	25.07.2013	DCM	1429	1.13	1262	na	na	na	na	na
DRA	1874	22.07.2013	DCM	2538	1.41	1799	03.10.2013	DCM	1261	2.25	560
SES	1657	22.07.2013	DCM	1174	1.29	908	03.10.2013	DCM	1023	1.50	682
OBB	1311	18.07.2013	DCM	1511	1.25	1210	na	na	na	na	na
BREN	1590	18.07.2013	DCM	982	1.59	616	07.10.2013	DCM	474	1.45	327
WIS	1180	16.07.2013	DCM	5039	11.77	428	10.10.2013	DCM	5113	8.64	592
PIB	913	15.07.2013	DCM	1904	8.92	214	10.10.2013	DCM	2857	7.54	379
LAS	851	10.07.2013	DCM	2531	1.32	1916	16.10.2013	DCM	3017	2.14	1410
BAR	567	10.07.2013	DCM	2579	3.95	653	na	na	na	na	na
SOS	2799	na	na	na	na	na	14.10.2013	CWS	933	7.58	123
GKS	2413	25.07.2013	CWS	832	1.81	460	01.10.2013	CWS	1102	2.64	418
OPL	2344	25.07.2013	CWS	1150	1.17	985	01.10.2013	CWS	1020	1.27	801
DRA	1874	22.07.2013	CWS	2551	1.46	1749	03.10.2013	CWS	1779	2.54	700
SES	1657	22.07.2013	CWS	1314	1.46	898	03.10.2013	CWS	1160	1.68	689
OBB	1311	18.07.2013	CWS	1269	1.62	782	07.10.2013	CWS	666	0.89	748
BREN	1590	18.07.2013	CWS	1038	1.67	620	07.10.2013	CWS	715	1.49	481
WIS	1180	16.07.2013	CWS	3148	10.28	306	10.10.2013	CWS	5072	8.92	568
PIB	913	15.07.2013	CWS	2361	9.66	244	10.10.2013	CWS	3679	11.08	332
LAS	851	10.07.2013	CWS	2636	1.74	1515	16.10.2013	CWS	3025	2.15	1409
BAR	567	10.07.2013	CWS	2124	3.46	614	16.10.2013	CWS	2670	6.62	403

C:P, C:N, N:P are mean molar ratios. BAR: Baggersee Rossau, LAS: Lansersee, PIB: Piburgersee, WIS: Wildsee bei Seefeld, BREN: Brennersee, OBB: Obernbergersee, SES: Sebensee, DRA: Drachensee, OPL: Oberer Plenderlesee, GKS: Gossenköllesee, SOS: Schwarzsee ob Sölden. na: data not available.

### Stoichiometry of the microbial and seston fractions

The C:P ratios of the microbial fraction were in almost all cases higher than the Redfield ratio (Table 2). In most lakes, the microbial fraction was P-depleted relative to C in July (mean C:P: ~345) with the exception of OPL and Drachensee (DRA). In July, the microbial fraction of these two lakes exhibited a C:P very close to 106, whereas in October the microbial fraction of all lakes was poor in P compared to C. The microbial fraction of the four subalpine lakes situated at low elevation (i.e., 567–1180 m a.s.l.) and that of Gossenköllesee (GKS) and Sebensee (SES) was slightly N-depleted compared to Redfield (6.6). In July, the C:P ratios of the seston fraction showed the same trend as that of the microbial fraction. The seston of almost all lakes was P-depleted relative to C, especially in the subalpine lakes and in the alpine lakes GKS and SES. The C:N ratios in the subalpine lakes Baggersee Rossau (BAR), Lansersee (LAS), Piburgersee (PIB), and Wildsee (WIS), as well as in the alpine lakes GKS and SES showed a depletion, all other lakes again had a slightly lower C:N ratio than the Redfield Ratio (6.6). However, this trend was more pronounced in July than in October (Table 2). Water temperature was not significantly correlated with changes in stoichiometric ratios in the two fractions, except for the C:N ratio of the seston fraction in the CWS (Supplementary Fig. S1). Finally, the C:P ratio of the microbial and seston fractions was not significantly correlated with that of POC:chlorophyll-a (Supplementary Fig. S2) suggesting that detritus did not affect the trends observed.

**Table 2:** Stoichiometry of the microbial (MIC) and seston (SEST) fractions in composite water samples (CWS) and in samples from the deep chlorophyll-a maximum (DCM).

Lake	Sample	Fraction	Elevation (m a.s.l)	Date	C:P	C:N	N:P	Date	C:P	C:N	N:P
SOS	DCM	MIC	2799	na	na	na	na	14.10.2013	298	5	55
GKS	DCM	MIC	2413	25.07.2013	402	7	61	01.10.2013	248	7	37
OPL	DCM	MIC	2344	25.07.2013	113	2	62	na	na	na	na
DRA	DCM	MIC	1874	22.07.2013	175	4	45	03.10.2013	2262	5	467
SES	DCM	MIC	1657	22.07.2013	233	7	35	03.10.2013	3210	5	700
OBB	DCM	MIC	1590	18.07.2013	159	3	54	na	na	na	na
BREN	DCM	MIC	1311	18.07.2013	197	4	45	07.10.2013	335	5	65
WIS	DCM	MIC	1180	16.07.2013	432	6	78	10.10.2013	265	18	18
PIB	DCM	MIC	913	15.07.2013	429	11	41	10.10.2013	166	7	23
LAS	DCM	MIC	851	10.07.2013	456	7	61	16.10.2013	728	10	76
BAR	DCM	MIC	567	10.07.2013	163.6	7.4	22	na	na	na	na
SOS	CWS	MIC	2799	na	na	na	na	14.10.2013	1693	9	192
GKS	CWS	MIC	2413	25.07.2013	426	4	108	01.10.2013	475	5	92
OPL	CWS	MIC	2344	25.07.2013	86	2	42	01.10.2013	353	7	50
DRA	CWS	MIC	1874	22.07.2013	111	4	32	03.10.2013	586	6	95
SES	CWS	MIC	1657	22.07.2013	163*	11*	15*	03.10.2013	3032	5	671
OBB	CWS	MIC	1590	18.07.2013	296	5	63	07.10.2013	730	5	154
BREN	CWS	MIC	1311	18.07.2013	288	4	78	07.10.2013	392	5	81
WIS	CWS	MIC	1180	16.07.2013	443	8	64	10.10.2013	322	15	21
PIB	CWS	MIC	913	15.07.2013	474	8	62	10.10.2013	375	18	29
LAS	CWS	MIC	851	10.07.2013	783	7	110	16.10.2013	491	9	57
BAR	CWS	MIC	567	10.07.2013	376	9	40	16.10.2013	795	10	80
SOS	DCM	SEST	2799	na	na	na	na	14.10.2013	354	8	43
GKS	DCM	SEST	2413	25.07.2013	376	7	54	01.10.2013	235	7	32
OPL	DCM	SEST	2344	25.07.2013	284	5	57	na	na	na	na
DRA	DCM	SEST	1874	22.07.2013	144	5	30	03.10.2013	356	6	56
SES	DCM	SEST	1657	22.07.2013	349	14	26	03.10.2013	452	6	78
OBB	DCM	SEST	1590	18.07.2013	106	6	17	na	na	na	na
BREN	DCM	SEST	1311	18.07.2013	164	7	25	07.10.2013	212	6	37
WIS	DCM	SEST	1180	16.07.2013	385	11	37	10.10.2013	369	13	31
PIB	DCM	SEST	913	15.07.2013	320	9	37	10.10.2013	241	15	16
LAS	DCM	SEST	851	10.07.2013	427	8	53	16.10.2013	411	9	44
BAR	DCM	SEST	567	10.07.2013	424	10	42	na	na	na	na
SOS	CWS	SEST	2799	na	na	na	na	14.10.2013	499	8	63
GKS	CWS	SEST	2413	25.07.2013	440	7	62	01.10.2013	458	8	56
OPL	CWS	SEST	2344	25.07.2013	147	4	38	01.10.2013	371	7	52
DRA	CWS	SEST	1874	22.07.2013	212	5	45	03.10.2013	627	9	73
SES	CWS	SEST	1657	22.07.2013	118	5	22	03.10.2013	674	6	114
OBB	CWS	SEST	1590	18.07.2013	250	6	42	07.10.2013	1015	5	194
BREN	CWS	SEST	1311	18.07.2013	179	6	32	07.10.2013	229	6	36
WIS	CWS	SEST	1180	16.07.2013	318	7	44	10.10.2013	525	7	77
PIB	CWS	SEST	913	15.07.2013	324	7	46	10.10.2013	311	11	28
LAS	CWS	SEST	851	10.07.2013	756	14	54	16.10.2013	317	9	34
BAR	CWS	SEST	567	10.07.2013	440	10	43	16.10.2013	394	9	41

C:P, C:N, N:P are mean molar ratios. * indicates only one replicate. BAR: Baggersee Rossau, LAS: Lansersee, PIB: Piburgersee, WIS: Wildsee bei Seefeld, BREN: Brennersee, OBB: Obernbergersee, SES: Sebensee, DRA: Drachensee, OPL: Oberer Plenderlesee, GKS: Gossenköllesee, SOS: Schwarzsee ob Sölden. na: data not available.

### Bacterial abundance and community composition

Bacterial abundance in the CWS and DCM samples ranged from 1.92 × 10^5^ cells mL^−1^ to 2.80 × 10^6^ cells mL^−1^ in July and from 1.85 × 10^5^ cells mL^−1^ to 2.62 × 10^6^ cells mL^−1^ in October (Supplementary Fig. S3). In July, lake bacterial abundance decreased steadily along the elevational gradient, but in October this trend was not so obvious.

The bacterial assemblage was dominated by *Actinobacteria* in seven out of eleven lakes in October (Supplementary Fig. S4). By contrast, in July *Betaproteobacteria* was the dominant group in five out of ten lakes or co-dominated the bacterial assemblage with *Actinobacteria* in two other lakes. In July, relative abundance of *Bacteroidetes* was between 10–25% of DAPI-stained cell in lakes situated at intermediate and high elevation (WIS, BREN, OBB, SES, DRA, OPL, GKS), whereas in October their relative abundance decreased notably in the alpine lakes SES, DRA, OPL, and GKS (Supplementary Fig. S4). Generally, *Alphaproteobacteria* accounted for less than 9% of DAPI-stained cells in all lakes with the exception of SOS in October, where their relative abundance reached 21% of DAPI counts. Probe GAMMA 42a targeting *Gammaproteobacteria* was only used in the lowest lakes to increase the coverage of bacterial community with the group-specific probes. However, *Gammaproteobacteria* represented <1% of DAPI counts in those lakes and only in LAS in October, their relative abundance reached 3% of DAPI-stained cells (Supplementary Fig. S4). In July, a significant positive relationship between the relative abundance of R-BT *Betaproteobacteria* and of *Bacteroidetes* and lake elevation was found (Supplementary Fig. S5).

### Comparison of regressions

**Figure 1:**
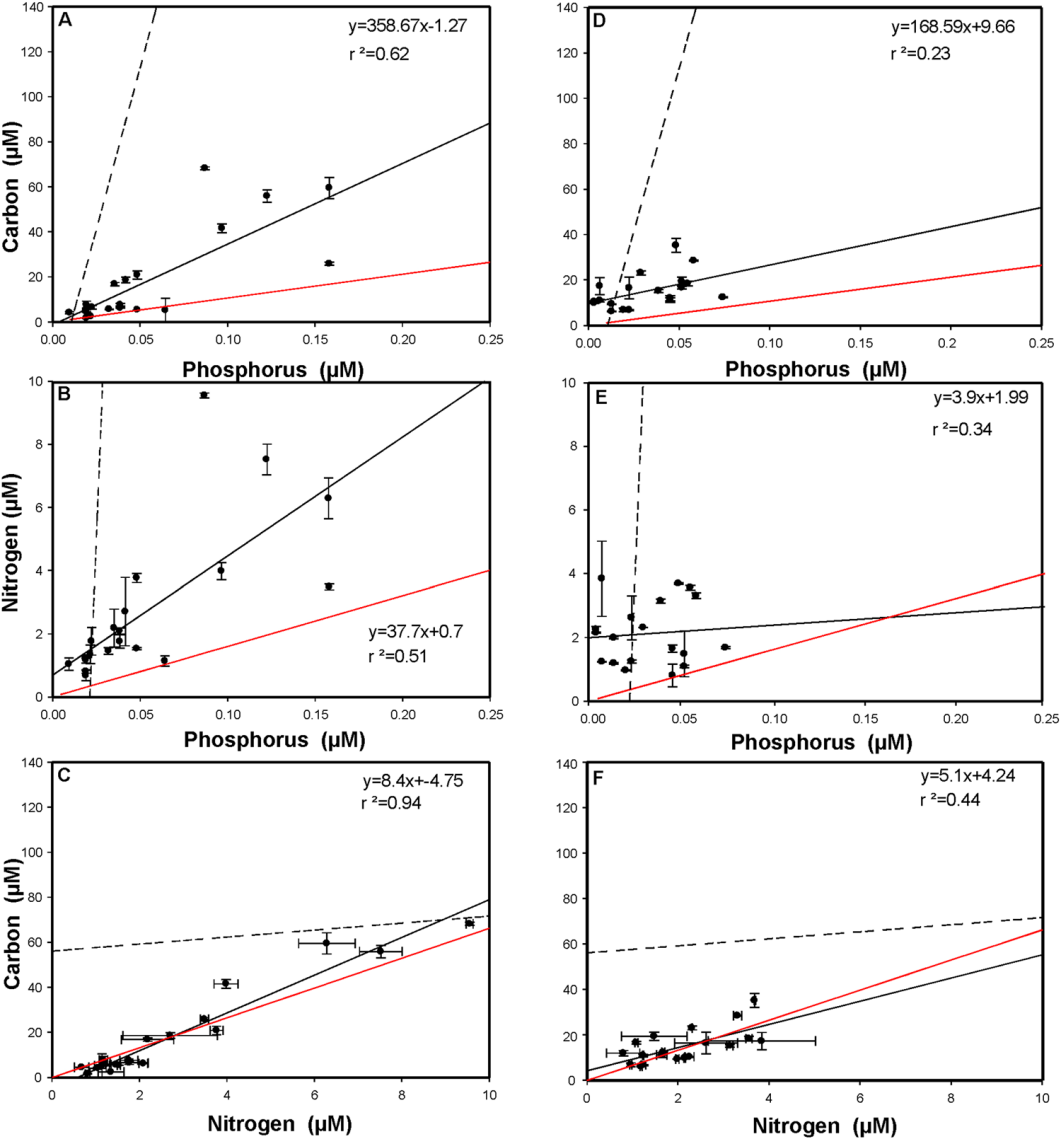
Regression analysis between the concentration of carbon, nitrogen, and phosphorus in the microbial size-fraction (0.7–1 µm) in July (**A**,**B**,**C**) and in October (**D**,**E**,**F**). The red line represents the Redfield ratio of these elements, the continuous line represents the regression for the microbial size-fraction (with corresponding equation) and the dotted line that for the pooled dissolved fraction.

**Figure 2:**
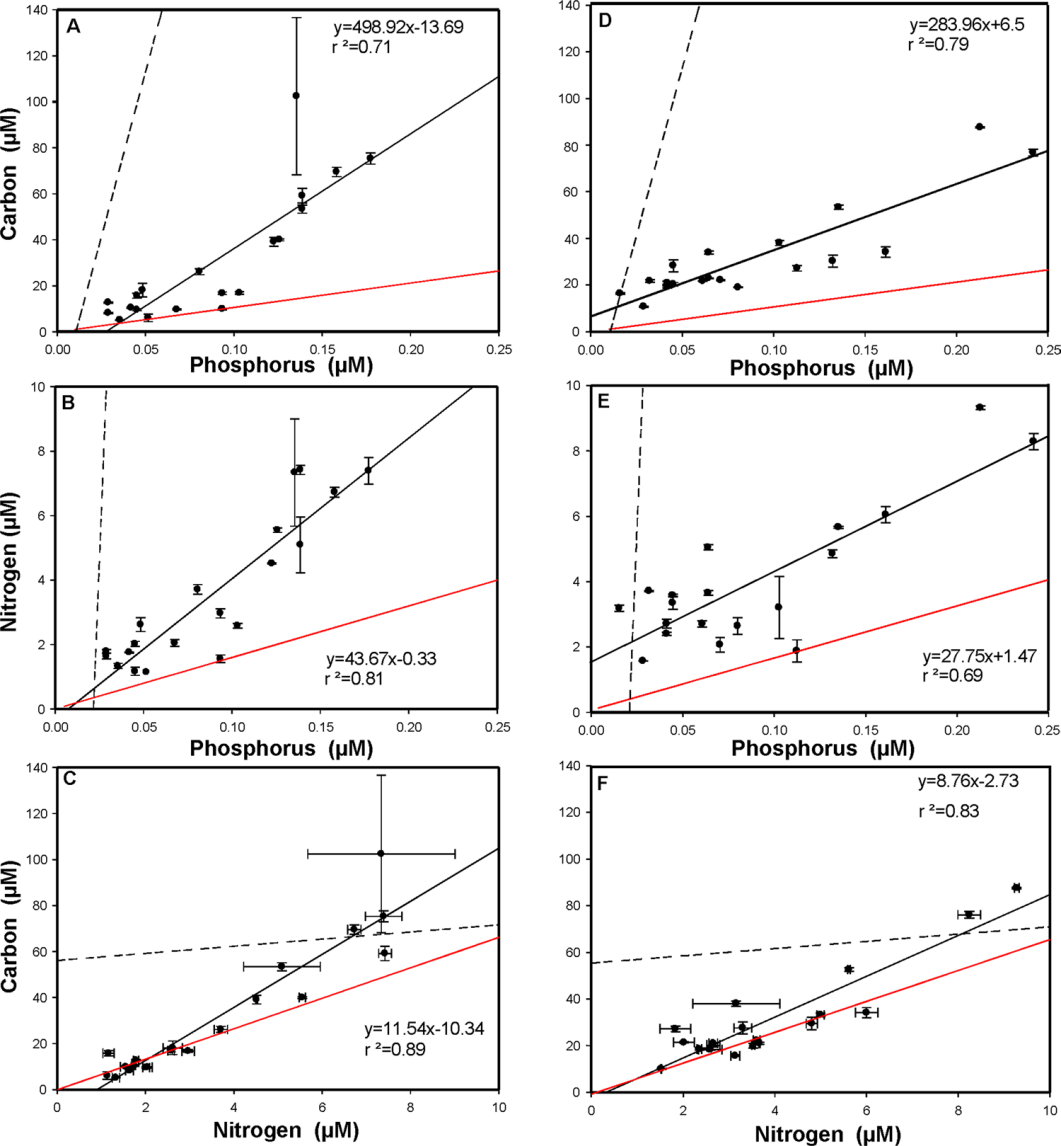
Regression analysis between the concentration carbon, nitrogen, and phosphorus in the seston size (0.7–50 µm) fraction in July (**A**,**B**,**C**) and in October (**D**,**E**,**F**). The red line represents the Redfield ratio of these elements, the continuous line represents the regression of the seston fraction (with corresponding equation) and the dotted line that for the pooled dissolved fraction.

The slopes and intercepts of the regressions between C and P, N and P, as well as C and N for the two types of water samples (CWS and DCM) did not significantly differ (one-way ANCOVA) in July except for the slope of the N versus C in the seston fraction. Thus, the data from both types of samples were pooled together and a new linear regression was calculated for each fraction and sampling period (Fig. 1 and Fig. 2, Supplementary Table S3). A significant difference in the slope and intercept of the C versus N relationship between the seston and microbial fraction was found in July (Supplementary Table S3). In October, the slope of the N versus P and of C versus N relationship differed significantly between the pooled microbial and pooled seston fraction (Supplementary Table S3). The relationship between C and P, N and P, and C and N were described by significantly different regression lines in July as compared to October for both the seston and the microbial fraction (Fig. 1, Fig. 2 and Supplementary Table S3), but not for the dissolved fraction (Supplementary Table S3).

## Discussion

The main objective of this study was to assess whether the stoichiometry of the microbial fraction, usually dominated by bacteria^10^, differs among lakes situated along an elevational (trophic) gradient reflecting changes in the stoichiometry of the dissolved fraction (resource). Several factors such as water temperature and cell growth rate influence bacterial stoichiometry, however, resource elemental composition is supposed to be the most important one^10, 27, 28^. We found that dissolved nutrient concentrations differed greatly among lakes (Supplementary Table S2). As expected, subalpine lakes had higher absolute dissolved nutrient and DOC concentrations. However, in those lakes the nutrient ratios of the dissolved fraction, particularly the C:P ratios, were as high or even higher than in most of the alpine lakes (Table 1), for example, in Baggersee Rossau (567 m a.s.l) was 2124:1 in July versus 832:1 in Gossenköllesee (2413 m a.s.l.). The reason for this unexpected result is mainly the disproportionally high DOC concentrations in the subalpine lakes as compared to their P concentrations. DOC concentrations in the subalpine lakes situated at lower elevations were on average, one order of magnitude higher than in the alpine lakes, whereas TDP concentrations were 3–7 times higher. In the littoral zone of some of the subalpine lakes, emergent macrophytes were prevalent. These primary producers might contribute to the production of autochthonous DOM that are usually enriched in carbon as compared to other nutrients^29^. Our results are also in agreement with those of Kopáček *et al*.^25^, where higher dissolved C:P ratios were found in lakes with forested catchments (DOC:TN:TP ratio of 983:75:1) than in those situated above the treeline (DOC:TN:TP ratio of 302:77:1). Although the C:P ratios we found were higher than those in the study of Kopáček *et al*.^25^, they support the trend observed by these authors.

The main processes contributing to external nutrient inputs to lakes are the export from the terrestrial catchment and atmospheric deposition. Nutrients transported through the atmosphere can originate both far or near the lakes. For oligotrophic alpine lakes, long distance sources such as soil-derived dust from desert regions, combustion sources, agriculture, and industry are very important^30, 31^. By contrast, local P sources such as pollen, plant fragments, primary biogenic aerosols such as for example, bacterial detritus, can be relevant for lakes situated in forested areas^32, 33^. A possible explanation for the different dissolved nutrient ratios found between lakes situated above and below treeline are changes in the relative importance of the mentioned external nutrient sources and a differential retention of nutrients in the soil of the catchment surrounding the lakes^33^. The leaching of phosphorus retained in soils is often associated with DOC leaching, which is low for alpine catchments due to poor soil development and to low microbial activities^33^. Thus, terrestrial export in subalpine lakes is characterized by higher C:P ratios than in alpine lakes. Further, the relative importance of terrestrial nutrient sources decreases across the elevational gradient, whereas that of atmospheric deposition, characterized by lower C:P ratios, tend to increase with elevation^33, 34^. Thus, atmospheric nitrogen and phosphorus pollution could further alter the stoichiometry of alpine lakes in the future^35^.

While the C:P ratios of all lakes in our study were well above the Redfield ratio (Fig. 1. and Fig. 2), the C:N ratios in the dissolved fraction (except for the lakes Wildsee bei Seefeld and Piburgersee) were below it and showed less variability than the corresponding C:P ratios. This indicates that most of the lakes considered were enriched in N and only some of the subalpine lakes showed a slight relative nitrogen depletion.

Although the dissolved fraction was more P-depleted relative to C in the subalpine lakes, the microbial fraction was only more P-depleted in lakes below the treeline during the summer stratification, whereas in autumn no trend was related to the elevation of the lakes. This is probably due to more production and competition with autotrophs in summer. Nevertheless, it seems that the microbial fraction stoichiometry does not directly reflect the changes in the stoichiometry of the dissolved fraction occurring along the elevational gradient. This pattern could be masked by a temperature effect because average values were higher for subalpine than for alpine lakes and this could in fact affect bacterial growth and relative growth rates. However, we did not find a significant relationship between water temperature and the elemental ratios of the microbial fraction (Supplementary Fig. 1). This is probably because natural bacterial communities are locally adapted to the *in situ* water temperatures^36^.

The fact that C:P and N:P ratios of the microbial fraction were always lower than those of the dissolved fraction has important consequences for the function bacteria have in these lakes. Bacteria may constrain primary production in P-limited ecosystems because of their competitive advantage in taking up inorganic P and act therefore, rather as sink than as link for nutrients^10^. Stoichiometrically flexible bacteria have the capacity of switching from competitors to regenerators through feedback mechanisms that affect phytoplankton production and indirectly the available carbon resources used by bacteria^27, 37^. Bacteria can regulate their elemental composition via homeostatic control within a much more narrow range than is possible in their natural environment^22, 27^. However, P-limited bacteria are neither P–rich nor invariant in nutrient content, but rather adapt their elemental composition to the element currently limiting (C or P)^28^. In fact, individual bacterial populations can switch from biomass C:P homeostasis in P-depleted surroundings to no homeostasis under nutrient replete conditions^28, 37^. In some of the studied lakes, the C:P ratio of the dissolved fraction was 11-fold higher than the C:P ratio of the microbial fraction and a similar trend was observed for N:P ratios. Only in Sebensee in October, the N:P ratio of the dissolved fraction showed a slightly lower value than that of the microbial fraction. This suggests that most lakes in our study present P limitation, no matter at which elevation they are located.

Microbes cope with nutrient limitation following mainly two strategies^38^ and this have different effects on stoichiometric strategies, i.e., homeostoichs vs. heterostoichs^37^. One strategy (competition specialists) is based on the “growth rate hypothesis” (GRH)^9^, and focuses on the increase of growth rates and consequently of the assembly machinery. Fast growing cells increase the production of nucleic acid and ribosomes containing N and P, which are needed to achieve high protein synthesis levels. Another possible strategy (i.e., uptake specialist) is to focus on resource acquisition machinery which corresponds to nutrient uptake proteins which contain N and C, but less P^39^. In natural bacterial communities, the observed stoichiometry probably results from the sum of the multiple strategies exhibited by individual bacterial populations ranging from strict to no homeostasis^28, 37^. Further studies are needed to identify which strategy of stoichiometric regulation is used by the dominant bacterial populations in alpine and subalpine lakes.

Finally, another important factor to consider when analyzing the stoichiometry of the microbial fraction is the diversity of this fraction by itself. Different bacterial groups might have different uptake abilities for diverse nutrients and exhibit different degrees of homeostasis^27, 28^. At present, we lack an appropriate method that allows assessing concomitantly the taxonomy of bacteria and their elemental composition. Nevertheless, we observed some interesting trends in the composition of the bacterial community across the elevational gradient. Unfortunately, those trends could not be directly related to changes in the microbial fraction stoichiometry. The bacterial assemblage of our study lakes was co-dominated by *Actinobacteria* and *Betaproteobacteria*. We found that *Actinobacteria* generally dominated the bacterial community at both sampling dates, but *Betaproteobacteria* was the second most abundant group in most lakes. This is in agreement with previous studies in alpine lakes^40, 41^ and in other freshwater systems^42–44^. *Betaproteobacteria* are considered as a fast growing group^45^. Thus, we expected that due to their fast growth rates and according to the GRH, the microbial fraction of lakes dominated by this group could be P richer than that of systems dominated by other groups. Among *Betaproteobacteria*, the R-BT subgroup is the one that exhibits the highest growth rates, with doubling times as short as 16 h^23, 46^. These doubling times are the highest reported for a freshwater bacterial group. In July, we observed a significant increase of the relative importance of the R-BT subgroup with elevation. However, the C:P ratio of the microbial fraction did not always decrease accordingly and the microbial fraction of the alpine lake Gossenköllesee, where the R-BT bacteria represented a substantial part of the bacterial community (up to 33%) had a C:P ratio as high as that of Piburgersee, where R-BT represented a smaller fraction of the bacterial assemblage.

In summary, given the pivotal role of heterotrophpic bacteria in the nutrient cycles of aquatic ecosystems, our results strengthen the idea of heterotrophic bacteria being a ‘sink’ for phosphorus, as in all studied lakes they tended to immobilize phosphorus, whereas they might act as nitrogen mineralizers (‘link’). Nevertheless, the stoichiometry of the microbial fraction showed a large variability among mountain lakes with C:P and N:P ratios well above the Redfield ratio. Thus, our results further suggest that bacterial assemblages may not be as nutrient-rich as compared to other food-web components, but instead have to be seen as spatio-temporal dynamic in their stoichiometry. While our work could link these changes to the resource itself, understanding how stoichiometric changes in the microbial fraction relate to bacterial community composition will need further studies.

## Methods

### Sampling sites and main characteristics

To assess changes in the stoichiometry of the different fractions along an elevational gradient, lakes distributed above and below the treeline (range: 567–2799 m a.s.l.) were selected. Lakes situated above the treeline or alpine lakes have usually small catchments areas composed of bare rocks with poorly developed soils and scarce vegetation^47^. Alpine lakes are at the very end of the trophic scale and have very low DOC (<1000 µg L^−1^) and phosphorus concentrations (<4.0 µg L^−1^). The second group of lakes sampled, consisted of subalpine lakes. Those have larger catchment areas surrounded by trees or meadows with well-developed soils and vegetation. In consequence, DOC and nutrient concentrations are higher than in alpine lakes. These lakes show a lower water transparency and higher water temperatures^47, 48^. Overall, 7 subalpine lakes and 4 alpine lakes were sampled, although one of the alpine lakes, Schwarzsee ob Sölden (SOS), was only sampled in autumn. The main characteristics of the sampling sites are summarized in Supplementary Table S1.

### Sampling

Samples were collected in July 2013 during the summer stratification and in October, when most of the lakes had already undergone the autumn overturn. During each sampling period, the lakes were sampled within two weeks of each other to minimize temporal variability. Water samples were collected from a boat over the deepest point of the lake using a modified 5-L Schindler-Patalas sampler (5 L). Before samples were collected, all containers and bottles were washed with diluted HCl (0.1 N) and rinsed thoroughly with MQ water (Millipore) and then, with water from the respective lake. Temperature, pH, conductivity, dissolved oxygen, and chlorophyll-a (i.e., relative fluorescence) were recorded with a YSI 6600-V 2 multiparameter probe. Two different water samples were collected in every lake, namely, a water sample (10 L) from the deep chlorophyll-a maximum (if it was detectable) and a composite sample (10 L) from the whole water column (i.e., equal volume collected at 1 m depth intervals, n = 5–17 depending on maximum depth). Water from the deep chlorophyll-a maximum (DCM) was collected to determine whether the stoichiometry of the microbial and seston fractions at this depth differed from that of the average for the water column. The depth of the chlorophyll-a maximum was assessed with the YSI 6600 V2 probe equipped with a ‘chlorophyll’ sensor which detects the fluorescence emitted by chlorophyll-a upon excitation with blue light (470 nm LED).

### Size fractionation of water samples

Water collected from the DCM and the composite water sample (CWS) were separated into three different size fractions defined operationally by the type of filter used: the dissolved fraction (<0.7 µm), the microbial fraction (0.7–1 µm) and the seston fraction (0.7–50 µm). All filtrations were done in the laboratory in the shortest possible time (hours) following sampling. A 50 µm nylon mesh net was used to pre-screen the whole water and to obtain the seston fraction. To collect the microbial fraction, the lake water was then pre-filtered through a 1 µm acid-rinsed polycarbonate filter (47 mm, Millipore). Subsequently, each filtrate (seston and microbial fraction) was filtered onto double pre-ignited (3 h at 450 °C) glass-fiber filters (GF/F filters, 25 mm, Whatman with a retention efficiency of 0.7 µm) to collect the biomass for the analysis of their carbon and nitrogen content with a CHN analyzer. The selection of GF/F filters for defining the “dissolved” fraction was done according to Godwin & Cotner^37^. Due to the different particle load of every lake, different volumes were concentrated onto the GF/F filters. The exact volume of the filtrate was recorded for every fraction and used for later calculations. After the filtration steps, the filters were dried at 60 °C for at least 48 h. All filtrations were run in duplicate.

### Water chemistry

All water samples were collected in prewashed polyethylene containers and stored at 4 °C till chemical analyses were run (within 24 h). To assess the stoichiometry of the dissolved fraction, we measured dissolved organic carbon (DOC), total dissolved nitrogen (TDN) and total dissolved phosphorus (TDP). DOC and TDN concentrations were measured with a total organic carbon analyzer (Shimadzu TOC-Vc series) equipped with a TNM−1 nitrogen module. For the analyses, 30 mL of the sample were filtered through a pre-treated (4 h at 450 °C) glass-fiber filter (GF/F filters, Whatman) and collected into a pre-treated glass bottle (4 h at 450 °C), acidified to pH 2 with HCl, and stored at 4 °C in the dark until the analyses were made. Both parameters (DOC and TDN) were detected simultaneously after combustion and catalytic oxidation of the injected sample. Calibration and comparison with a consensus reference material was done as described elsewhere^49^. Before TDP analyses were made, samples were filtered through pre-treated GF/F filters, as above. The TDP concentration was assessed by spectrophotometry using the molybdate blue method after digestion of the sample with sulfuric acid and hydrogen peroxide^50^.

### Stoichiometry of the seston and microbial size fraction

For particulate organic carbon (POC) and nitrogen (PON) analyses, the dried GF/F filters were analyzed with a Flash EA 1112 (Thermo Fisher Scientific) CHN analyzer. Empty tin capsules and unused pre-treated GF/F filters were used as blanks. For calibration, acetanilide was used at five different concentrations. Total particulate phosphorus (TPP) content of the microbial and seston fractions was calculated by subtracting the total dissolved phosphorus concentration (TDP) in the GF/F filtrate from the total phosphorus concentration (TP) in either the <1 filtrate or the <50 filtrate (Supplementary Table S4). The TP concentration of the seston and microbial filtrates was determined using the molybdate blue method^50^. To test whether detritus could have an effect on the seston and microbial C:P ratios observed, we used the POC to chlorophyll-a ratio as proxy^51^. The chlorophyll-a values used in this test were those obtained from spectrophotometric measurements (acetone extraction) done as described elsewhere^49^.

### Bacterial abundance in whole and size-fractioned samples

Immediately after sampling, aliquots from the whole and pre-filtrated (<1 µm) water were taken. Samples to estimate bacterial abundance were fixed with 0.2 µm pre-filtered formaldehyde (2% final concentration). Later on, 5 to 10 mL sub-samples were filtered onto black polycarbonate filters (GTTP, 0.2 µm, Millipore) and stained with DAPI (4′,6′-diamidino-2-phenylindole)^52^. For DAPI counting, an epifluorescence microscope (Zeiss Axioplan) was used. To determine bacterial abundance, 400 to 500 cells were counted in 20–40 randomly chosen microscopic fields. Because the GF/F filters have a retention efficiency defined as 0.7 µm, but no precise pore size, some bacteria might pass through those filters. Therefore, we assessed bacterial abundance in the filtrate obtained after filtration through single and double GF/F filters. Bacterial cell losses due to the filtration through a single GF/F filter represented on average 7.2% of bacterial abundance, but only 5.2% when two filters were used (Supplementary Fig. S6). We also checked for possible bacterial cell losses in the <1 µm filtrate as compared to the respective whole water sample, however, bacterial abundance was not significantly different (df = 21; p > 0.05).

### Bacterial community structure (CARD-FISH)

Catalyzed reported deposition–fluorescence *in situ* hybridization (CARD-FISH) was used to assess the bacterial community structure^53^. Whole water aliquots were fixed with formaldehyde (2% final concentration), filtered onto white polycarbonate filters (GTTP, 0.2 µm, Millipore) and stored at −20° until analysis. Thawed filters were embedded in 0.1% agarose to prevent cell loses and permeabilized according to Sekar *et al*.^54^. Six group-specific 5′-horseradish peroxidase (HRP) labeled oligonucleotide probes (ThermoFisher-Hybaid) were used in this study to get an overview of composition of the bacterial community. Probes targeting *Bacteria* (EUBI-III), *Alphaproteobacteria* (ALF968), *Betaproteobacteria* (BET42a), R-BT subgroup of *Betaproteobacteria* (R-BT065), Ac-I lineage of *Actinobacteria* (AcI-852), *Bacteroidetes* (CF319a), and *Gammaproteobacteria* (GAM42a) were used. To exclude the possibility of a false targeting, we tested this latter probe with a positive control (*E. coli* culture) that confirmed that the probe worked properly.

Filter sections were placed in 0.5 mL reaction vials containing HRP-labeled probe and the hybridization buffer (formamide concentration 55%.) in a 1:100 ratio. Hybridizations were run for 2:15 hour in a hybridization oven set at 35 °C. Afterwards, the samples were washed first in a preheated washing buffer (10 min at 37 °C) containing the appropriate NaCl concentration and then in 1x PBS buffer (10 min, RT). The signal–amplification step was run for 30 min. For this purpose, filter sections were incubated at 37° with a substrate mix containing the amplification buffer and Alexa Fluor 488 tyramide in a 1:100 ratio. Following the amplification step, filter sections were washed with 1x PBS (15 min, RT and dark), MQ water (10 min, RT) and 96% ETOH. Subsequently the filter sections were dried and mounted on slides using an anti-fading solution containing DAPI (1 µg mL^−1^). All CARD-FISH preparations were examined using a Zeiss Axioplan microscope equipped with a 100 W Hg lamp and filter-sets 1 and 9 for DAPI and Alexa 488, respectively. A minimum of 350 DAPI-stained cells were counted on each filter section. Probe-specific positive cells were recorded from the same microscopic field as DAPI-stained cells. Thus, all CARD-FISH results are expressed as percentage of DAPI-stained cells.

### Statistical analyses

One-way ANCOVA was used to compare the slopes and intercepts of the linear regressions obtained between elements (C vs N, C vs P and N vs P) in the microbial, seston and dissolved fraction. The elemental concentrations of P, N, and C were plotted against each other and a linear regression model was fitted to the data. To check for differences in bacterial abundance in filtrates obtained after filtration through GF/F and 1 µm polycarbonate filters to whole water, a paired t-test was used. All statistical analyses were run using the free PAST software.

## Electronic supplementary material

Supplementary Information Stenzel et al.
