# Supplementary material for: Stoichiometry of natural bacterial assemblages from lakes located across an elevational gradient
Source: Sci Rep. 2017 Jul 19;7:5875. doi: 10.1038/s41598-017-06282-0 (PMC5517659; doi:10.1038/s41598-017-06282-0)
Supplement: Supplementary file 1 — Supplementary Information Stenzel et al. [file 41598_2017_6282_MOESM1_ESM.pdf]

Supplementary Information

Stoichiometry of natural bacterial assemblages from lakes located across an elevational gradient

*Birgit Stenzel, Carina Rofner, Maria Teresa Pérez, and Ruben Sommaruga**

Institute of Ecology, Lake and Glacier Ecology Research Group, Technikerstraße 25, University of Innsbruck, 6020 Innsbruck, Austria

Correspondence and requests for materials should be addressed to R.S. (ruben.sommaruga@uibk.ac.at)

Supplementary Table S1. Geographic coordinates, elevation, maximum depth (Z_{max}), lake and catchment area. The dashed line indicates the separation between subalpine and alpine lakes. BAR (Baggersee Rossau), LAS (Lansersee), PIB (Piburgersee), WIS (Wildsee bei Seefeld), BREN (Brennersee), OBB (Obernbergersee), SES (Sebensee), DRA, (Drachensee) OPL (Oberer Plenderlesee), GKS (Gossenköllesee) and SOS (Schwarzsee ob Sölden). na: data not available.

Lake	Latitude (N)	Longitude (E)	Elevation (m a.s.l)	Z_{max} (m)	Lake area (ha)	Catchment area (ha)
BAR	47°15´	11°26´	567	14	2.8	na
LAS	47°14´	11°25´	851	10.4	3.5	na
PIB	47°11´	10°53´	913	24.6	13.4	265
WIS	47°19´	11°11´	1180	5.5	6.1	690
BREN	47°01´	11°30´	1311	9.2	5.5	na
OBB	46°59´	11°24´	1590	9.7	12	1163
SES	47°21´	10°56´	1657	16	6.4	na
DRA	47°02´	10°56´	1874	19.5	4.5	188
OPL	47°12´	11°02´	2344	7	2.1	97
GKS	47°13´	11°00´	2413	9.9	1.7	30
SOS	46°57´	10°56´	2799	18	3.5	18

Supplementary Table S2. Summary of the water chemistry analyses, water temperature and chlorophyll-a (extraction) during the July and October sampling. DCM (deep chlorophyll maximum), CWS (composite water sample), total dissolved phosphorus (TDP), total dissolved nitrogen (TDN), dissolved organic carbon (DOC), chlorophyll-a (Chl-a), and water temperature (Temp.: mean of CWS). na: data not available. The dashed line indicates the separation between subalpine and alpine lakes. BAR (Baggersee Rossau), LAS (Lansersee), PIB (Piburgersee), WIS (Wildsee bei Seefeld), BREN (Brennersee), OBB (Obernbergersee), SES (Sebensee), DRA, (Drachensee) OPL (Oberer Plenderlesee), GKS (Gossenköllesee), and SOS (Schwarzsee ob Sölden).

Lake	Elevation (m a.s.l.)	Sample	Date	TDP ($\mu g\ L^{-1}$)	DOC ($\mu g\ L^{-1}$)	TDN ($\mu g\ L^{-1}$)	Temp. (C°)	Chl-a ($\mu g\ L^{-1}$)	Date	TDP ($\mu g\ L^{-1}$)	DOC ($\mu g\ L^{-1}$)	TDN ($\mu g\ L^{-1}$)	Temp. (C°)	Chl-a ($\mu g\ L^{-1}$)
SOS	2799	na	na	na	na	na	na	na	14.10.2013	0.90	325	50.02	3.64	2.18
SOS	2799	na	na	na	na	na	na	na	14.10.2013	0.90	335	51.02	3.64	1.67
GKS	2413	DCM	25.07.2013	0.60	273	190.09	11.25	2.83	01.10.2013	1.20	351	179.09	7.21	4.18
GKS	2413	CWS	25.07.2013	0.90	290	187.09	11.25	2.31	01.10.2013	0.90	384	170.08	7.21	1.67
OPL	2344	DCM	25.07.2013	0.30	166	171.08	7.69	1.54	na	na	na	na	na	na
OPL	2344	CWS	25.07.2013	0.40	178	178.09	7.69	1.35	01.10.2013	0.60	237	217.10	5.07	na
DRA	1874	DCM	22.07.2013	0.70	393	325.16	7.48	0.71	03.10.2013	1.00	488	253.12	7.45	2.60
DRA	1874	CWS	22.07.2013	0.70	395	316.15	7.48	0.51	03.10.2013	0.80	551	253.12	7.45	2.17
SES	1657	DCM	22.07.2013	0.40	318	287.14	6.02	2.64	03.10.2013	1.00	396	308.15	5.89	5.12
SES	1657	CWS	22.07.2013	0.40	356	284.14	6.02	2.44	03.10.2013	1.00	449	311.15	5.89	3.21
BREN	1590	DCM	18.07.2013	0.60	418	306.15	11.77	0.51	07.10.2013	nd	459	369.18	8.26	0.84
BREN	1590	CWS	18.07.2013	0.90	442	308.15	11.77	0.51	07.10.2013	0.90	498	391.19	8.26	na
OBB	1311	DCM	18.07.2013	1.10	351	328.16	10.30	1.29	na	2.50	na	na	na	na
OBB	1311	CWS	18.07.2013	1.10	442	318.15	10.30	1.09	07.10.2013	1.80	238	304.15	9.08	4.24
WIS	1180	DCM	16.07.2013	1.50	2928	290.14	16.55	4.11	10.10.2013	1.50	2971	401.19	11.01	3.02
WIS	1180	CWS	16.07.2013	2.40	2927	332.16	16.55	3.28	10.10.2013	1.50	2947	385.18	11.01	2.96
PIB	913	DCM	15.07.2013	2.80	2065	270.13	10.01	3.02	10.10.2013	1.80	1992	308.15	10.31	2.57
PIB	913	CWS	15.07.2013	2.50	2287	276.13	10.01	2.44	10.10.2013	1.50	2137	225.11	10.31	2.51
LAS	851	DCM	10.07.2013	5.00	4903	4329.07	14.56	na	16.10.2013	4.40	5143	2802.34	11.69	5.91
LAS	851	CWS	10.07.2013	5.30	5413	3628.74	14.56	na	16.10.2013	4.40	5157	2800.34	11.69	5.34
BAR	567	DCM	10.07.2013	3.00	2997	885.42	18.71	na	na	na	na	na	na	na
BAR	567	CWS	10.07.2013	3.30	2715	915.44	18.71	na	16.10.2013	3.50	3620	637.30	13.99	na

Supplementary Table S3. Summary of the one-way ANCOVA analyses used to compare regression lines. Numbers are p values: pooled composite and deep chlorophyll maximum samples (CC), composite water sample (CWS), deep chlorophyll maximum (DCM), MIC (microbial fraction), SEST (seston fraction), DISS (dissolved fraction), CCJO (pooled composite and deep chlorophyll maximum sample July and October). Significance values (<0.05) are indicated in bold.

**Compared Data**	**P values intercept and slope**	**C *vs* P**	**N *vs* P**	**C *vs* N**
JULY MIC (CWS-DCM)	intercept	0.2751	0.4306	0.7892
	slope	0.1091	0.2437	0.8084
JULY SEST (CWS-DCM)	intercept	0.1461	0.0948	0.6856
	slope	0.2253	0.5497	**0.0052**
OCT MIC (CWS-DCM)	intercept	0.1560	0.3910	0.5709
	slope	0.0844	0.3091	0.5336
OCT SEST (CWS-DCM)	intercept	0.1477	0.1635	0.5930
	slope	0.1072	0.1378	0.4435
JULY DISS (CWS-DCM)	intercept	0.5923	0.5687	0.7720
	slope	0.7257	0.5376	0.4096
OCT DISS (CWS-DCM)	intercept	0.6739	0.8823	0.8464
	slope	0.8174	0.3980	0.9125
JULY MIC (CC)-SEST (CC)	intercept	0.5061	0.0843	**0.0366**
	slope	0.0649	0.4061	**3.82E-05**
OCT MIC (CC)-SEST (CC)	intercept	0.5045	0.1509	0.3787
	slope	0.1190	**0.0192**	**0.0216**
JULY MIC (CC)-OCT MIC (CC)	intercept	0.1363	0.9448	0.1801
	slope	**0.0476**	**0.0114**	**0.0117**
JULY SEST (CC)-OCT SEST (CC)	intercept	0.7223	0.1573	0.2093
	slope	**0.0006**	**0.0019**	**0.0025**
JULY DISS (CC)- OCT DISS (CC)	intercept	0.9814	0.3901	0.5303
	slope	0.1797	0.9885	0.1256
DISS (CCJO)-MIC (CC) JULY	intercept	**1.568E-09**	**1.13E-05**	**0.0060**
	slope	**2.34E-18**	**4.43E-14**	0.09
DISS (CCJO)-MIC (CC) OCT	intercept	**6.77E-05**	**0.035**	**0.0037**
	slope	**3.691E-11**	**1.211E-07**	0.72
DISS (CCJO)-SEST (CC) JULY	intercept	**1.136E-13**	**7.546E-09**	**0.0436**
	slope	**3.95E-17**	**2.95E-14**	**0.017**
DISS (CCJO)-SEST (CC) OCT	intercept	**6.56E-05**	**5.67E-07**	**0.048**
	slope	**1.79E-29**	**1.41E-16**	0.140

Supplementary Table S4. Summary of the measured and calculated phosphorus concentrations in the microbial (MIC) and seston (SEST) fractions of composite water samples (CWS) and of samples from the deep chlorophyll maximum (DCM). Total dissolved phosphorus (TDP), total phosphorus in filtrates (TP), PP (calculated particulate P), na: data not available. BAR (Baggersee Rossau), LAS (Lansersee), PIB (Piburgersee), WIS (Wildsee bei Seefeld), BREN (Brennersee), OBB (Obernbergersee), SES (Sebensee), DRA, (Drachensee) OPL (Oberer Plenderlesee), GKS (Gossenköllesee), and SOS (Schwarzsee ob Sölden).

Lake	Sample	Fraction	Date	TDP ($\mu g\ L^{-1}$)	TP ($\mu g\ L^{-1}$)	PP ($\mu g\ L^{-1}$)	Date	TDP ($\mu g\ L^{-1}$)	TP ($\mu g\ L^{-1}$)	PP ($\mu g\ L^{-1}$)
SOS	CWS	MIC		na	na	na	14.10.2013	0.90	1.30	0.40
GKS	CWS	MIC	25.07.2013	0.90	1.20	0.30	01.10.2013	0.90	1.10	0.20
OPL	CWS	MIC	25.07.2013	0.40	1.00	0.60	01.10.2013	0.60	1.20	0.60
DRA	CWS	MIC	22.07.2013	0.70	2.20	1.50	03.10.2013	0.80	1.50	0.70
SES	CWS	MIC	22.07.2013	0.40	2.40	2.00	03.10.2013	1.00	1.10	0.10
OBB	CWS	MIC	18.07.2013	0.90	1.50	0.60	07.10.2013	0.90	1.30	0.40
BREN	CWS	MIC	18.07.2013	1.10	1.80	0.70	07.10.2013	1.80	3.00	1.20
WIS	CWS	MIC	16.07.2013	2.40	3.70	1.30	10.10.2013	1.50	3.10	1.60
PIB	CWS	MIC	15.07.2013	2.50	3.60	1.10	10.10.2013	1.50	3.10	1.60
LAS	CWS	MIC	10.07.2013	5.30	8.00	2.70	16.10.2013	4.40	6.20	1.80
BAR	CWS	MIC	10.07.2013	3.30	8.20	4.90	16.10.2013	3.50	4.40	0.90
SOS	DCM	MIC		na	na	na	14.10.2013	0.90	1.60	0.70
GKS	DCM	MIC	25.07.2013	0.60	1.20	0.60	01.10.2013	1.20	2.60	1.40
OPL	DCM	MIC	25.07.2013	0.30	1.00	0.70	na	na	na	na
DRA	DCM	MIC	22.07.2013	0.70	1.70	1.00	03.10.2013	1.00	1.20	0.20
SES	DCM	MIC	22.07.2013	0.40	1.00	0.60	03.10.2013	1.00	1.10	0.10
OBB	DCM	MIC	18.07.2013	0.60	1.80	1.20	na	na	na	na
BREN	DCM	MIC	18.07.2013	1.10	2.30	1.20	07.10.2013	2.50	4.20	1.70
WIS	DCM	MIC	16.07.2013	1.50	3.00	1.50	10.10.2013	1.50	2.90	1.40
PIB	DCM	MIC	15.07.2013	2.80	5.80	3.00	10.10.2013	1.80	4.10	2.30
LAS	DCM	MIC	10.07.2013	5.00	8.80	3.80	16.10.2013	4.40	5.90	1.50
BAR	DCM	MIC	10.07.2013	3.00	7.90	4.90	16.10.2013	na	na	na
SOS	CWS	SEST		na	na	na	14.10.2013	0.90	2.20	1.30
GKS	CWS	SEST	25.07.2013	0.90	1.80	0.90	01.10.2013	0.90	2.20	1.30
OPL	CWS	SEST	25.07.2013	0.40	1.50	1.10	01.10.2013	0.60	1.50	0.90
DRA	CWS	SEST	22.07.2013	0.70	2.10	1.40	03.10.2013	0.80	2.20	1.40
SES	CWS	SEST	22.07.2013	0.40	2.00	1.60	03.10.2013	1.00	2.00	1.00
OBB	CWS	SEST	18.07.2013	0.90	2.20	1.30	07.10.2013	0.90	1.40	0.50
BREN	CWS	SEST	18.07.2013	1.10	4.00	2.90	07.10.2013	1.80	5.90	4.10
WIS	CWS	SEST	16.07.2013	2.40	6.30	3.90	10.10.2013	1.50	3.50	2.00
PIB	CWS	SEST	15.07.2013	2.50	5.00	2.50	10.10.2013	1.50	3.70	2.20
LAS	CWS	SEST	10.07.2013	5.30	9.50	4.20	16.10.2013	4.40	11.90	7.50
BAR	CWS	SEST	10.07.2013	3.30	8.20	4.90	16.10.2013	3.50	7.70	4.20
SOS	DCM	SEST		na	na	na	14.10.2013	0.90	2.80	1.90
GKS	DCM	SEST	25.07.2013	0.60	2.10	1.50	01.10.2013	1.20	3.70	2.50
OPL	DCM	SEST	25.07.2013	0.30	1.20	0.90	na	na	na	na
DRA	DCM	SEST	22.07.2013	0.70	2.80	2.10	03.10.2013	1.00	3.00	2.00
SES	DCM	SEST	22.07.2013	0.40	1.80	1.40	03.10.2013	1.00	2.40	1.40
OBB	DCM	SEST	18.07.2013	0.60	3.50	2.90	na	na	na	na

BREN	DCM	SEST	18.07.2013	1.10	4.30	3.20	07.10.2013	2.50	7.50	5.00
WIS	DCM	SEST	16.07.2013	1.50	5.80	4.30	10.10.2013	1.50	4.70	3.20
PIB	DCM	SEST	15.07.2013	2.80	6.60	3.80	10.10.2013	1.80	5.30	3.50
LAS	DCM	SEST	10.07.2013	5.00	9.30	4.30	16.10.2013	4.40	11.00	6.60
BAR	DCM	SEST	10.07.2013	3.00	8.50	5.50	16.10.2013	na	na	na

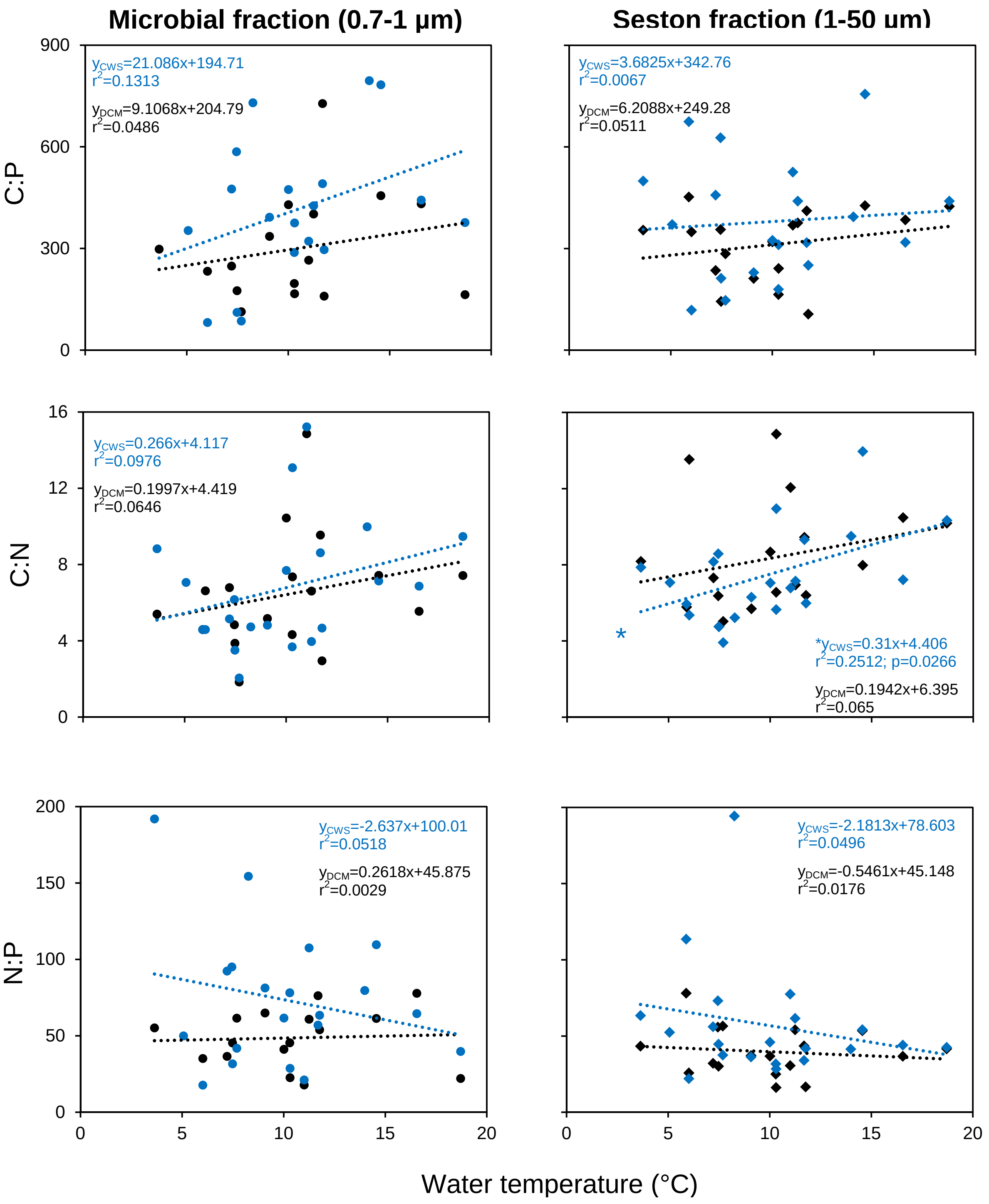

Supplementary Fig. S1. Regression of C:P, C:N and N:P ratios versus water temperature in the microbial and seston fraction. Blue dots and regression lines are for composite water samples (CWS), whereas black dots and regression lines represent samples taken from the deep chlorophyll maximum (DCM). A significant relationship between water temperature and the elemental ratios was only found for the C:N ratio of the seston fraction in the CWS, as indicated by an asterisk.

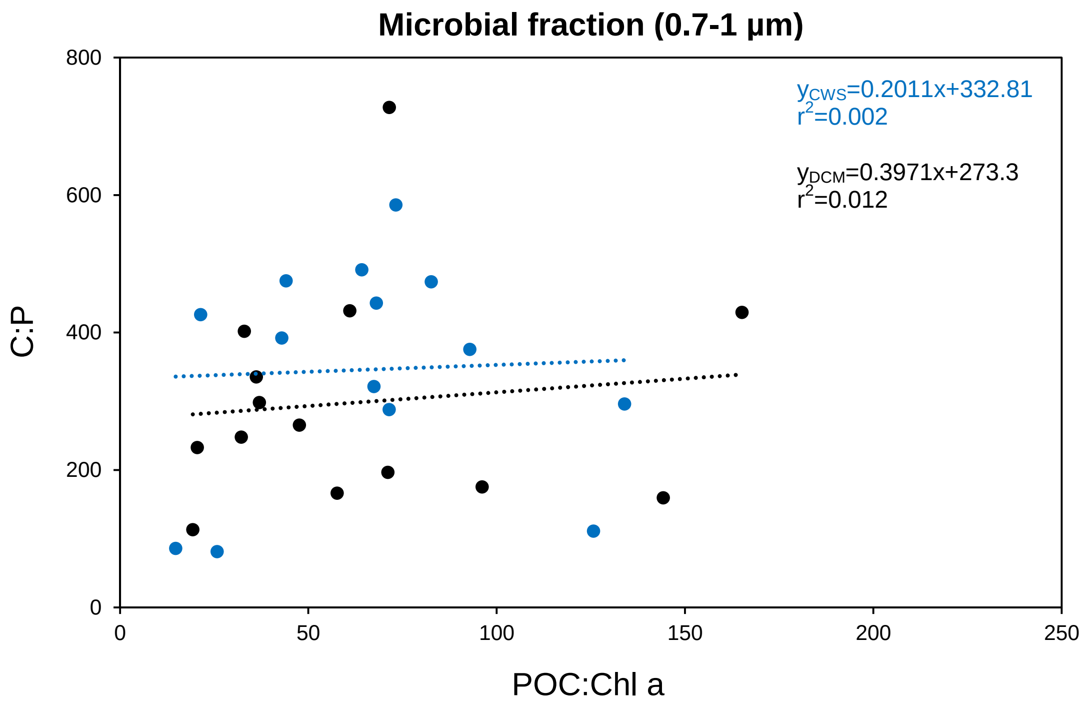

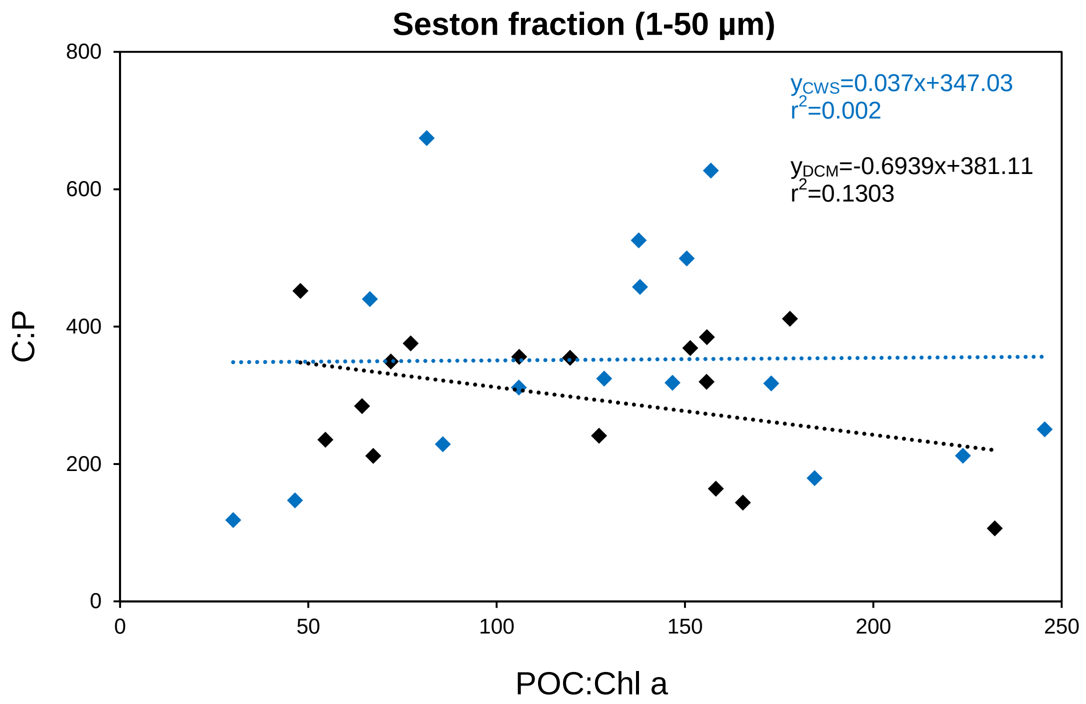

Supplementary Fig. S2. Relationship between C:P and the POC:Chl-ratio (as a proxy for the importance of detritus) in the microbial and seston fraction. Blue dots and regression lines represent composite water samples (CWS), whereas those in black represent samples taken from the deep chlorophyll maximum (DCM).

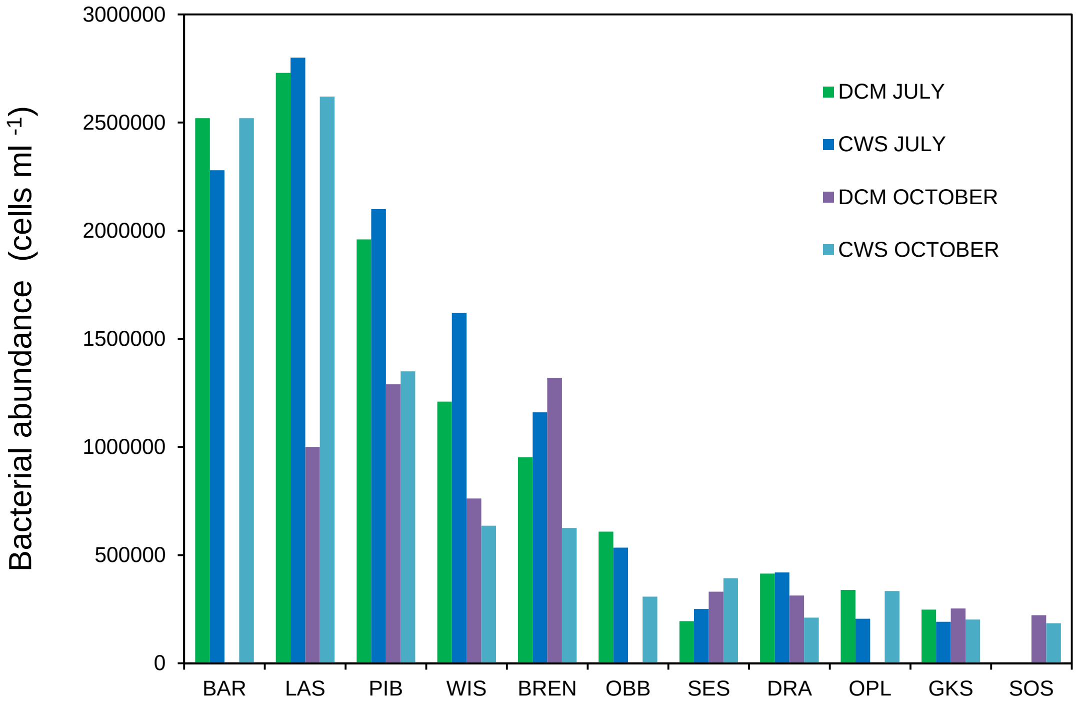

Supplementary Fig. S3. Bacterial abundance in July and October in composite water samples (CWS) and in those from the deep chlorophyll maximum (DCM). The lakes are ordered according to their elevation. In October, a chl-a max was not present in lakes BAR, OBB, and OPL. BAR (Baggersee Rossau), LAS (Lansersee), PIB (Piburgersee), WIS (Wildsee bei Seefeld), BREN (Brennersee), OBB (Obernbergersee), SES (Sebensee), DRA, (Drachensee) OPL (Oberer Plenderlesee), GKS (Gossenköllesee), and SOS (Schwarzsee ob Sölden).

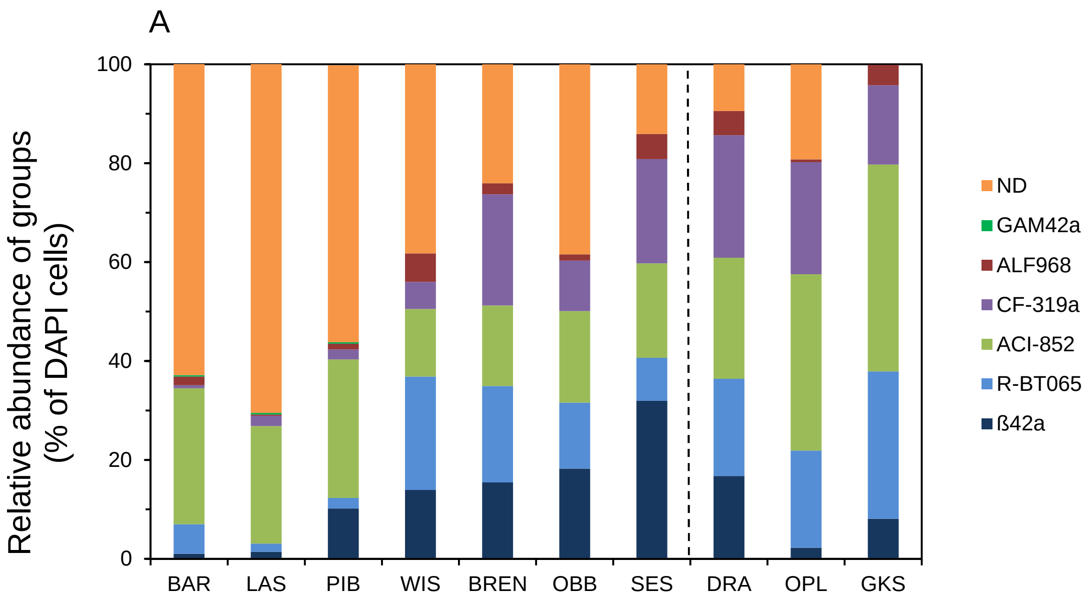

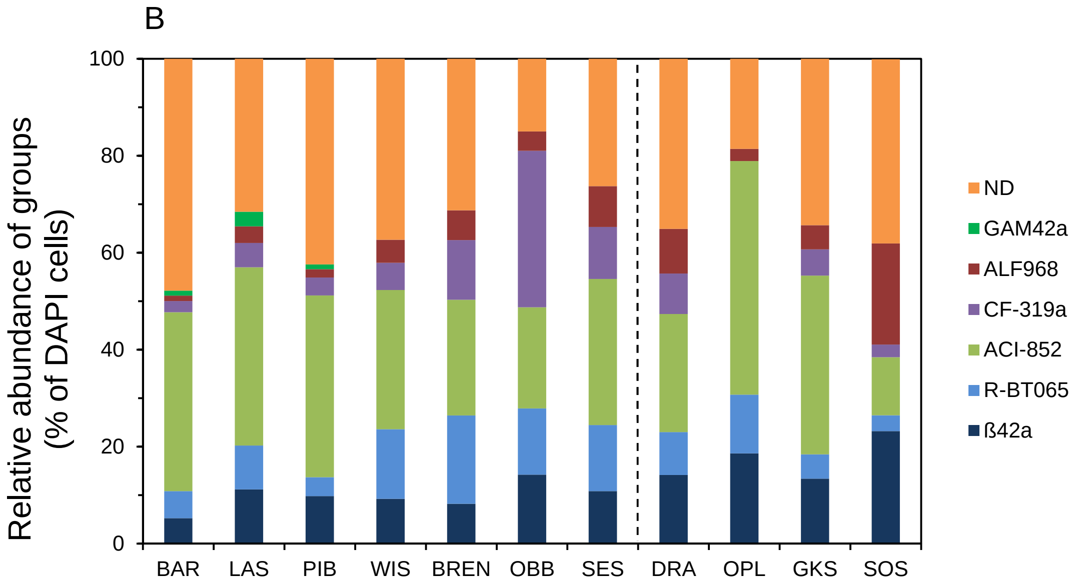

Supplementary Fig. S4. Structure of the bacterial community in July (A) and in October (B). The relative abundance of major bacterial groups is expressed as the percentage of DAPI-stained cells. The lakes are ordered according to their elevation and the dashed line indicates the separation between subalpine and alpine lakes. ALF968 (*Alphaproteobacteria*), BET42a (*Betaproteobacteria*), R-BT065 (R-BT subgroup of *Betaproteobacteria*), AcI-852 (Ac-I lineage of *Actinobacteria*), CF319a (*Bacteroidetes*), GAM42a (*Gammaproteobacteria*), ND (not defined). BAR (Baggersee Rossau), LAS (Lansersee), PIB (Piburgersee), WIS (Wildsee bei Seefeld), BREN (Brennersee), OBB (Obernbergersee), SES (Sebensee), DRA, (Drachensee) OPL (Oberer Plenderlesee), GKS (Gossenköllesee), and SOS (Schwarzsee ob Sölden).

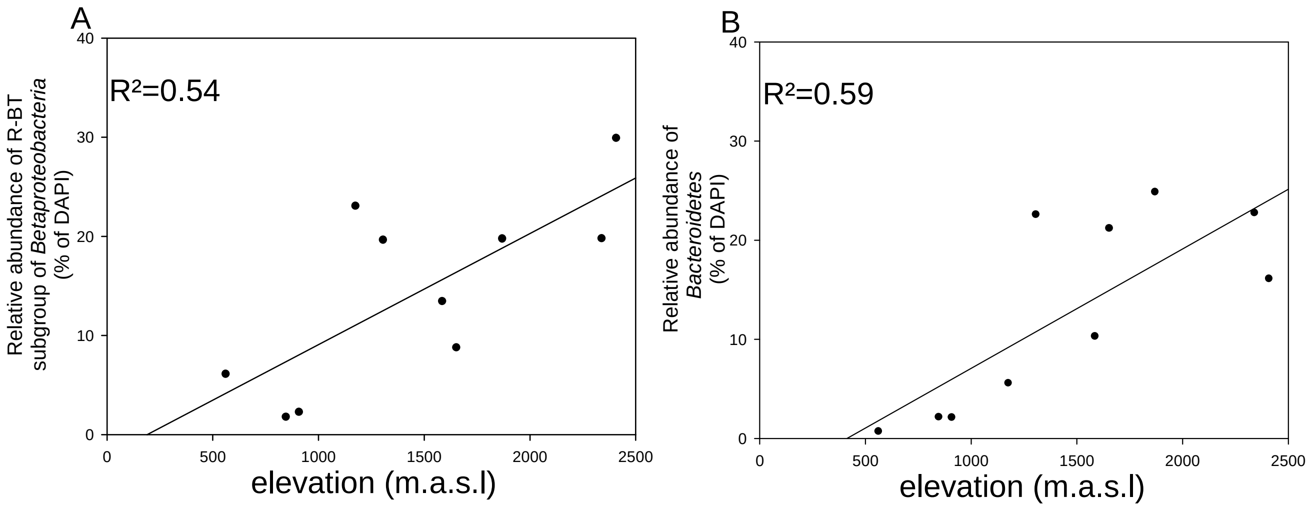

Supplementary Fig. S5. Relationship between the relative abundance of (A) R-BT bacteria and (B) *Bacteroidetes* and elevation for July.

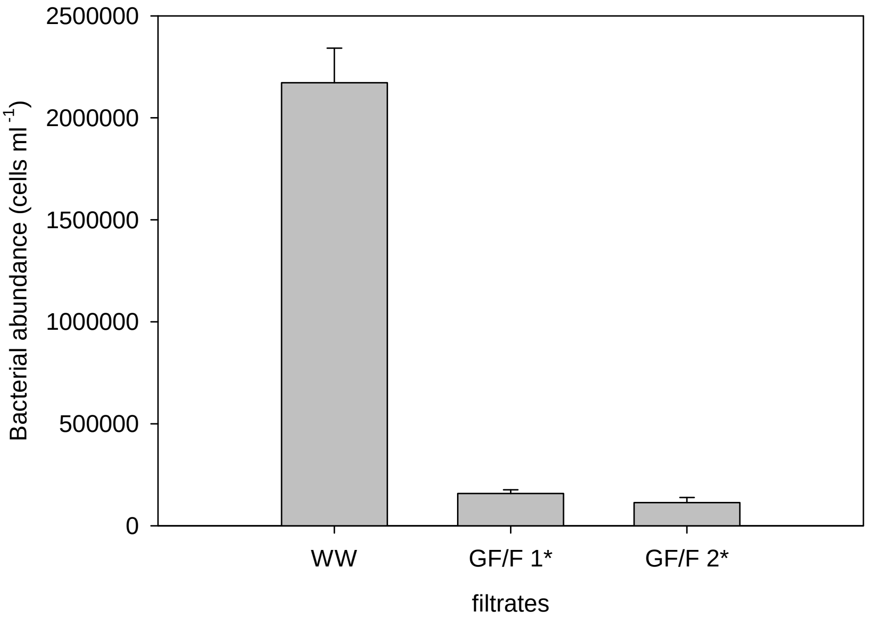

Supplementary Fig. S6. Bacterial abundance in the whole water (WW) and in samples obtained after filtration through single and double GF/F filters.
